# miR-26b Targets CEP135 Gene to Regulate Nasopharyngeal Carcinoma Proliferation and Migration by NF-κB Pathway

**DOI:** 10.1007/s12033-023-00691-5

**Published:** 2023-02-23

**Authors:** Guangrun Yang, Jiafu Zhou, Zhong Guo, Lixia Fan, Bowen Chen, Dapeng Zhang, Haitao Wen

**Affiliations:** 1 Department of Radiotherapy, The Third Affiliated Hospital of Qiqihar Medical University, Qiqihar City, China; 2 Department of Otolaryngology, The Third Affiliated Hospital of Qiqihar Medical University, Tiefeng District, 27 Taishun Street, Qiqihar City 161000, China

**Keywords:** Nasopharyngeal carcinoma, Hsa-miR-26b, CEP135, NF-κB pathway

## Abstract

To screen microRNAs (miRNAs) and analyze their role in the nasopharyngeal carcinoma (NPC) development through differential analysis and cytological validation of the nasopharyngeal carcinoma dataset. The Gene Expression Omnibus (GEO) database of NPC-related data were utilized to screen for differential miRNAs, downstream target genes and relevant pathways, and the relationships among them were verified by luciferase reporter assay and cell co-culture. To analyze the function of miRNAs and downstream target genes, a series of mimics, inhibitors or Small interfering RNAs (siRNAs) targeting the downstream target genes were transfected into NPC cells or normal epithelial cells by cell transfection techniques. Cell Counting Kit-8 (CCK8), Transwell, Enzyme-linked immunosorbent assay (ELISA) apoptosis, and western blotting were adopted to determine the changes in cell activity, invasiveness, and apoptosis after differential miRNA and target gene overexpression or downregulation. Differential analysis of miRNA dataset showed that the expression of miR-26b was significantly downregulated in NPC, in agreement with the validation results of nasopharyngeal carcinoma cell lines. And downregulation of miR-26b expression in normal nasopharyngeal epithelial cells transformed the cells to tumors. CEP135 was identified as the miR-26b downstream target gene by mRNA dataset analysis, and a luciferase reporter test revealed a direct targeting link between the two. Upregulation of CEP135 levels in nasopharyngeal cancer cell lines increased cell activity, accelerated cell migration, and inhibited apoptosis. The Kyoto Encyclopedia of Genes and Genomes (KEGG) enrichment analysis revealed that CEP135 exerted the above effects on cells via the NF-κB pathway, and co-culture with NF-κB pathway blockers reversed cell biological behavior to the level of the control group. MiR-26b downregulation leads to CEP135 overexpression and NF-κB pathway activation in NPC, which enhances proliferation, migration, and prevents apoptosis of nasopharyngeal carcinoma cells. Therefore, the study further clarifies the biological behavior mechanism of NPC and suggests new therapeutic options for NPC.

**Supplementary Information:**

The online version contains supplementary material available at 10.1007/s12033-023-00691-5.

## Introduction

Nasopharyngeal carcinoma (NPC) is one of the most frequent types of head and neck malignant tumors originating from epithelial cells [1], and is particularly prevalent in Guangdong, southern China and Southeast Asia [2]. The etiological factor of NPC is unclear, and studies have demonstrated that nasopharyngeal cancer may be related to a number of factors, including genetic susceptibility and Epstein-Barr virus (EBV) infection [3, 4]. NPC has unique genetic and biological behavioral features, with a high prevalence of distant metastasis being one of its negative biological behavior features. Seventy percent of patients had metastasis to the lymph nodes, whereas 20 percent had metastasized to the lung or liver, the most common sites [5]. As a primary therapeutic method for NPC, radiation therapy has been found to be the most effective [6]. However, distant metastatic events still happen even after radiotherapy and chemotherapy, indicating therapeutic failure [7]. In conclusion, much more in-depth research is required to fully identify the genetic mechanisms underlying tumor development and the genetic susceptibility mechanisms underlying adverse biological characteristics, such as susceptibility to metastasis in NPC [8, 9].

MicroRNAs (miRNAs), a class of small non-coding RNAs, play a regulatory role in gene expression and range in length from 18 to 24 nt [10]. MiRNA can inhibit gene expression by directly pairing with the base of target mRNA. MiRNAs have been discovered to be crucial in various cancers, including NPC [11–13]. In general, downregulated miRNAs target genes are highly expressed in cancer. These miRNAs can regulate the expression of target genes, thereby affecting the occurrence of cancer. For instance, miR-506 can prevent the metastasis and progression of NPC by downregulating LHX2, decreasing TCF4, and inhibiting Wnt/β-catenin signaling pathway [14]; miR-203a-3p targets LASP1 to prevent NPC development and metastasis [15]. miR-23a downregulation may be one of the mechanisms leading to NPC resistance to radiotherapy [16]. In addition, by targeting various downstream genes, miR-429 [17], miR-3188 and other miRNAs have a certain impact and predictive value on the treatment and prognosis of NPC patients [18]. These differential genes and downstream target pathways also provide a strategy for targeted therapy of NPC. However, only some miRNAs have been functionally studied so far, and the role of these miRNAs in tumor development, especially in NPC, is still in the initial stage of research. Therefore, a thorough understanding of miRNA gene regulation processes may give crucial prognostic markers as well as specific treatment options for NPC. However, only a few of these miRNAs have been investigated so far, and the role of these miRNAs in tumor development, particularly in NPC, is still in its infancy of investigation. As a result, a better knowledge of miRNA gene regulatory mechanisms may provide important prognostic biomarkers and individualized therapeutic targets for NPC.

In our study, we screened an NPC-related miRNA, miR-26b, using bioinformatics analysis of NPC-related data from public databases. The miR-26b is located in the fourth intron region of CTDSP1 gene. Previous researches have revealed that decreased miR-26b expression correlates with the progression of numerous tumors, such as colorectal cancer [19], lung cancer [20], and breast cancer [21]. Another member of the miR-26b family, miR-26a, has been connected to the formation of NPC [22], but the involvement of miR-26b in NPC has yet to be reported. We found that miR-26b expression downregulation could affect the proliferation, migration and apoptosis of NPC tumors in our study. Data analysis revealed that its downstream target gene, CEP135, was overexpressed, leading to aberrant activation of the NF-κB pathway, thus affecting the biological function of tumors. Our results elucidate the clinical relevance and functional mechanism of miR-26b in NPC, providing a new prognostic maker and therapeutically promising target for NPC patients.

## Methods

### Microarray Data Acquisition and Processing

The raw data for this study were collected from the Gene Expression Omnibus (GEO, https://www.ncbi.nlm.nih.gov/geo/), and the data acquired included two miRNA datasets for nasopharyngeal cancer (GSE32960 [23] and GSE43039 [24]) and one mRNA dataset (GSE12452 [25]). Among them, GSE32960 collected 330 samples (312 NPC samples and 18 samples from normal control) for a total of 916 miRNA expression matrices; GSE43039 obtained 40 samples (20 NPC cases and 20 cases from nasopharyngitis control) for a total of 800 miRNA expression matrices. The raw data were log2 transformed and then screened, and the missing value threshold for each sample was defined as 1.5 standard deviations over the negative control group’s mean. If more than 90% of the values were below the threshold, the miRNA was eliminated; if the missing values of the samples still had more than 60% after miRNA filtering, the samples were removed. The geometric mean was used to normalize the miRNA data. This is accomplished by employing a filtered data set in which the expression of each miRNA in each sample is multiplied by the sample’s geometric mean. For subsequent analysis, the log2 transformed data were used to transform the filtered geometric mean normalized data. The differentially expressed genes (DEGs) of GSE32960 and GSE43039 were analyzed using DEGs package in R software, with the following screening conditions were as follows: down-regulated DEGs were constructed defined as log2FoldChange (FC) less than -1 and adjusted P-value less than 0.05. The obtained down-regulated DEGs of GSE32960 and GSE43039 were used to construct a Venn diagram to display the overlapping areas. The TargetScan database (http://www.targetscan.org/) was used to estimate miRNA’s target genes, and the intersection with the results of subsequent analysis was carried out to clarify the downstream target genes of miRNAs.

### Gene Ontology (GO) and Kyoto Encyclopedia of Genes and Genomes (KEGG) Pathway Enrichment Analysis

GSE12452 obtained a total of 24,442 mRNA expression matrices from 41 samples (31 NPC cases and 10 normal cases). The Weighted Gene Co-expression Network (WGCNA) [26] was utilized to analyze the original expression matrices and filter relevant genes. Firstly, the clustering analysis was performed, and a power index of 5 was chosen as a suitable soft threshold to obtain a modular clustering map. Furthermore, enrichment analyses for the Kyoto Encyclopedia of Genes and Genomes (KEGG) [27] as well as Gene Ontology (GO) [28] were conducted on the genes of key modules obtained from the dataset. The downstream target genes of the miRNAs were discovered using R software’s “ggplot2” package analysis and context score ranking. Patients in GSE12452 dataset were separated into two groups based on the median expression of target genes: high expression and low expression. T-values of the whole genome were obtained by Bayesian analysis, and KEGG pathway enrichment analysis was plotted to find gene-related pathways.

### Cell Culture

CNE-1 and CNE-2 nasopharyngeal cancer cell lines were cultured [29, 30] in RPMI medium (Gibco) containing 10% fetal calf serum (FCS) (Gibco). The NP-69 nasopharyngeal epithelial cell line was cultured in specific KSFM (Invitrogen) medium containing 10% FCS with the addition of epidermal growth factor. All cell lines were cultivated in an incubator at 37 ℃ with 5% CO_2_, 21% O_2_, as well as 74% N_2_. The Cell Bank of the Chinese Academy of Sciences was where each and every one of the cell line strains was purchased (Shanghai, China).

### Reverse Transcription Quantitative PCR (RT-qPCR)

The expression of the genes to be studied in the cells was detected using RT-qPCR. The TRIzol reagent was utilized in order to get total RNA from the cultured cells (Invitrogen, Carlsbad, USA). SYBR Green (Qiagen) was used for the qPCR amplification process after a cDNA kit (Applied Biosytems) was used to produce reverse transcription of RNA (1000 ng) into cDNA. The other relative genes expression in some experiments were determine by the 2^−ΔΔCT^ method [31]. The PCR primers (RiboBio, Guangzhou, China) are as follows. miR-26b: Forward, 5′-CCGGGACCCAGTTCAAGTAA-3′, Reverse, 5′-CCCCGAGCCAAGTAATGGAG-3′, CEP-135: Forward, 5′-CGTCCCTCCGCGCCATTTTCA-3′, Reverse, 5′-CGGTATCCCAGCTGATCCAGCCTT-3′, GAPDH Forward, 5′-AGAAGGCTGGGGCTCATTTG-3′, Reverse, 5′-AGGGGCCATCCACAGTCTTC-3′

### Cell Transfection

Cells were passaged before transfection, 6-well plates were inoculated with 2 × 10^6^ cells per well and cultured to 70–80% confluence for the following experiments: (1) NP69 cells were divided into three groups and transfected, respectively, miR-26b inhibitor miRNA, miR-26b-inhibitor-NC (negative control), miR-26 (RiboBio, Guangzhou, China) and no transfection were used to observe the effect of miR-26b on normal nasopharyngeal epithelial cells; (2) CNE1 and CNE2 cells were divided into four groups and transfected, respectively, with miR-26b mimic, miR-26b inhibitor, miR-26b mimic-NC, and miR-26b inhibitor-NC (RiboBio, GuangZhou), in order to observe the effect of miR-26b on downstream target genes. The transfection procedure was performed by Lipofectamine 3000 reagent (Invitrogen; Thermo Fisher Scientific, Inc.), and the cells were collected 48 h after transfection for subsequent studies. The success of transfection was detected by RT-qPCR. The transfected miRNA sequence was UUCAAGUAAUUCAGGAUAGGU. Adenovirus containing CEP135 gene was transfected into CNE1 and CNE2 cell lines (GeneCopoeia, Guangzhou, China). Adenovirus-transfected cells were then screened with 2 g/ml of puromycin, and the expression of CEP135 was discovered using RT-qPCR.

### Dual-Luciferase Reporter Assay

Wild-type (WT) CEP135-3′UTR and mutation (MUT) is synthesized by Guangzhou Saicheng Biotechnology Co., Ltd. The 3′UTR of CEP135 was amplified by PCR to produce wild-type (WT); The 3′UTR of CEP135 was designed by point mutation and the mutant (MUT) was produced by PCR amplification. The wild-type (WT) CEP135-3′UTR and mutation (MUT) was cloned into the luciferase report vector (Gene Pharma, Shanghai) and co-transfected with hsa-miR-26b mimic or hsa-miR-NC. In 24-well plates, CNE-1 and CNE-2 cells underwent a 24-h incubation period. The cells were transfected, respectively, with 0.1 μg WT 3′UTR + 0.3 μg hsa-miR-26b mimic, 0.1 μg WT 3′UTR + 0.3 μg hsa-miR-NC, and 0.1 μg MUT 3′UTR + 0.3 μg hsa- miR-NC. A dual luciferase reporter assay system was used to look for luciferase luminescence activity after 48 h incubation (Promega, Madison, WI, USA). The relative luciferase activity was figured out by dividing the firefly luciferase luminescence by the Renilla luciferase luminescence. The experiment was carried out three times.

### Cell Proliferation Assay

The cell proliferation was measured by Cell Counting Kit-8 (CCK8) test. 1 × 10^3^ cells were put into each well of 96-well plates. On day 0 and day 5, 10 μl of CCK8 was introduced to each well, followed by a 2-h incubation at 37 °C. The samples’ absorbance values at 450 nm were obtained to depict the cell survival curve.

### Transwell Experiment

In 24-well plates, cells were injected at a cell concentration of 2 × 10^5^/ml cells. On a 24-well plate (Millipore, Billerica, MA, USA), 200 μl of cell suspension and 500 μl of medium containing 10% FCS were placed to the top and bottom, respectively. Following an incubation period of 48 h, the cells were preserved in methanol for 15 min, dyed with crystal violet for 20 min, and then counted under a microscope. Five randomly selected fields of view (40x) of invading cells were statistically analyzed.

### Enzyme-Linked Immunosorbent Assay (ELISA)

The ELISA kit (Roche Diagnostics Deutschland, Mannheim, Germany) was adopted to determine cell apoptosis. At room temperature, the cells were centrifuged at 1000 rpm and resuspended in 200 μl of cell lysis buffer for 30 min. After that, 20 μl supernatant was aspirated and was tested for nucleosome fragments in cell lysates as described in the ELISA kit.

### Western Blot

Cells were lysed and proteins were extracted using modified RIPA buffer and phenylmethylsulfonyl fluoride (PMSF, Beyotime, Shanghai, China). A Bicinchoninic acid (BCA) Protein Assay Kit was used to measure protein concentrations (Pierce, Rockford, IL, USA). Equal amounts of proteins were separated by 10% SDS-PAGE and transferred to polyvinylidene difiuoride (PVDF) membrane. The NF-κB primary antibody was incubated on the membranes for an entire night at 4 °C (CST, 55764 T). The membrane was washed with Tris buffered brine and incubated with secondary antibody for 2 h at room temperature. Protein bands were visualized using A chemiluminescence kit (Merck Millipore) was used to observe protein bands.

### Statistical Analysis

The R programming language was adopted to analyze the datasets obtained from the database. SPSS 22.0 was utilized to analyze all experimental data (IBM, Armonk, NY, USA). The data were expressed using the mean ± standard deviation (SD). Numerical variables for two independent samples were analyzed using *t*-test. Three or more groups of numerical variables were subjected to a one-way analysis of variance (ANOVA). When the results showed differences between groups, the SNK-q test was employed for two-by-two comparison between two groups. *P *< 0.05 was considered statistically significant.

## Results

### miR-26b Expression Is Downregulated in Nasopharyngeal Carcinoma

**Fig. 1:**
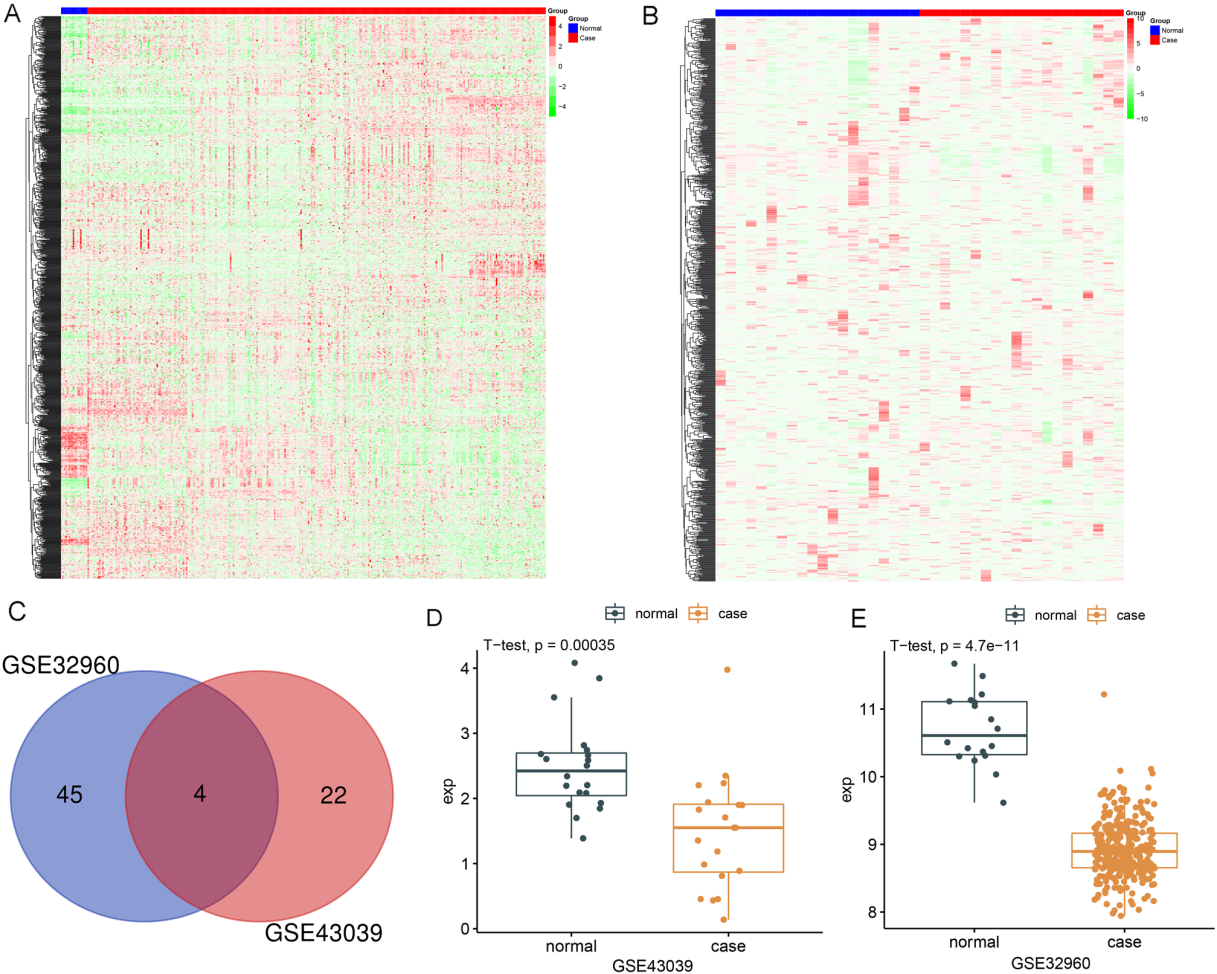
Bioinformatics analysis of nasopharyngeal carcinoma database to screen for low expression miRNAs in NPC. **A** Heatmap of differential expression gene in GSE32960; **B** Heatmap of differential expression gene in GSE43039; **C** Differential analysis of tumor samples and control samples in GSE32960 and GSE43039 to obtain low expression miRNAs; **D, E** In GSE32960 and GSE43039 NPC patients, hsa-miR-26 expression was considerably lower than in the control group (*P *< 0.05)

Two nasopharyngeal cancer miRNA datasets, GSE32960 and GSE43039, were downloaded from the GEO database, and heat maps of the two datasets were created using the “heatmap” package of R software (Fig. 1A, B). Differential analysis was done separately on the two datasets to identify low expression miRNAs, and the findings revealed 45 downregulated miRNAs in GSE32960 and 22 downregulated miRNAs in GSE43039. The intersection of the two was analyzed using a Venn diagram, and there were four miRNAs with downregulated expression in both datasets (Fig. 1C), which were hsa-miR-101/hsa-miR-26b/hsa-miR-143/hsa-miR-150. Because the function of hsa-miR-101/hsa-miR-143/hsa-miR-150 in the nasopharynx has already been reported, the study focuses on the functional mechanism of hsa-miR-26b in NPC cells. Validation of hsa-miR-26b expression in tumor and control samples revealed that hsa-miR-26b expression was considerably downregulated in nasopharyngeal carcinoma samples relative to controls (*P *< 0.01) (Fig. 1D, E). After analyzing the dataset, we discovered that hsa-miR-26b expression was downregulated in samples from NPC, which was confirmed in nasopharyngeal carcinoma cells.

### Downregulation of HSA-miR-26b Expression Induces Abnormal Proliferation of Normal Cells

**Fig. 2:**
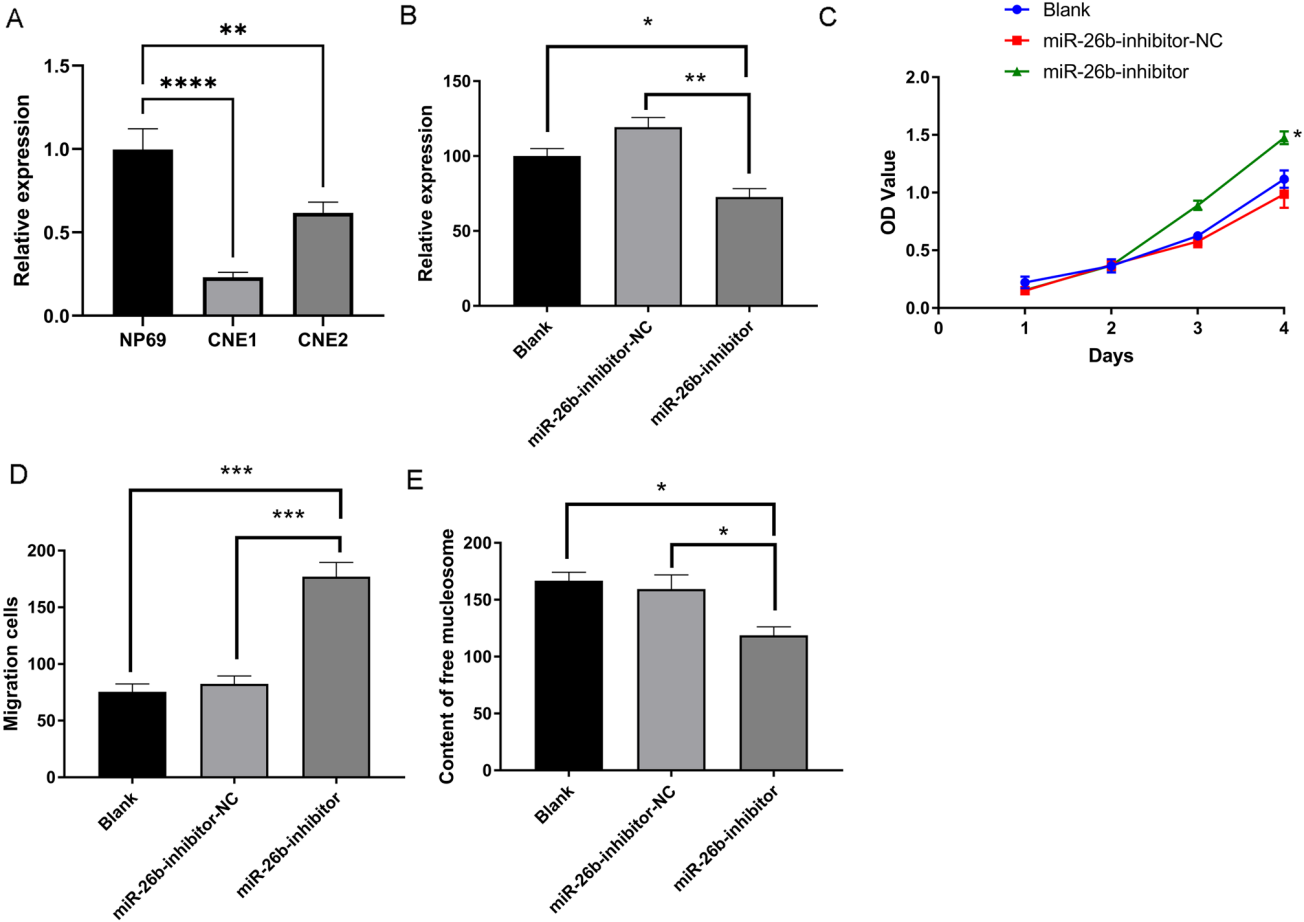
The role of hsa-miR-26b in the NPC. **A** hsa-miR-26b expression in CNE1, CNE2 compared with NP69; **B** NP69 cells were transfected with miR-26b-inhibitor and miR-26b-inhibitor-NC, and the expression level of miR-26b in each group was measured by RT-qPCR; **C** CCK8 cell proliferation assay among three groups; **D** Transwell assay among three groups; **E** ELISA of three groups (*P < 0.05, **P < 0.01, ***P < 0.001)

RT-qPCR was performed on CNE-1 and CNE-2 nasopharyngeal cancer cell lines and NP-69 nasopharyngeal normal epithelial cell line, and the results revealed that hsa-miR-26b expression was significantly decreased in CNE1 and CNE2 compared with that in NP69 (Fig. 2A). miR-26b-inhibitor and miR-26b-inhibitor-NC were transfected into NP69 cells to clarify the function of miR-26b expression downregulation in NPC development. It was discovered that the transfected miR-26b inhibitor group had lower levels of miR-26b expression than the other two control groups using RT-qPCR (Fig. 2B). The CCK8 assay revealed that the transfected miR-26b inhibitor group had considerably more proliferating live cells than the miR-26b-inhibitor-NC and non-transfected groups. The decrease in miR-26b expression accelerated the proliferation of NP69 cells (Fig. 2C). In addition, the results of Transwell assay showed that the migration cells in the miR-26b inhibitor group was significantly more than that in the two control groups, which was statistically significant (Fig. 2D). Furthermore, ELISA data demonstrated that the ELISA optical density values observed in cells transfected with miR-26b inhibitor were slightly lower than those measured in the other two control groups, but remained statistically significant (Fig. 2E). In conclusion, the down-regulation of miR-26b expression can promote cell proliferation and migration, and slow down apoptosis.

### CEP135 Is a Key Downstream Target of miR-26b in NPC

**Fig. 3:**
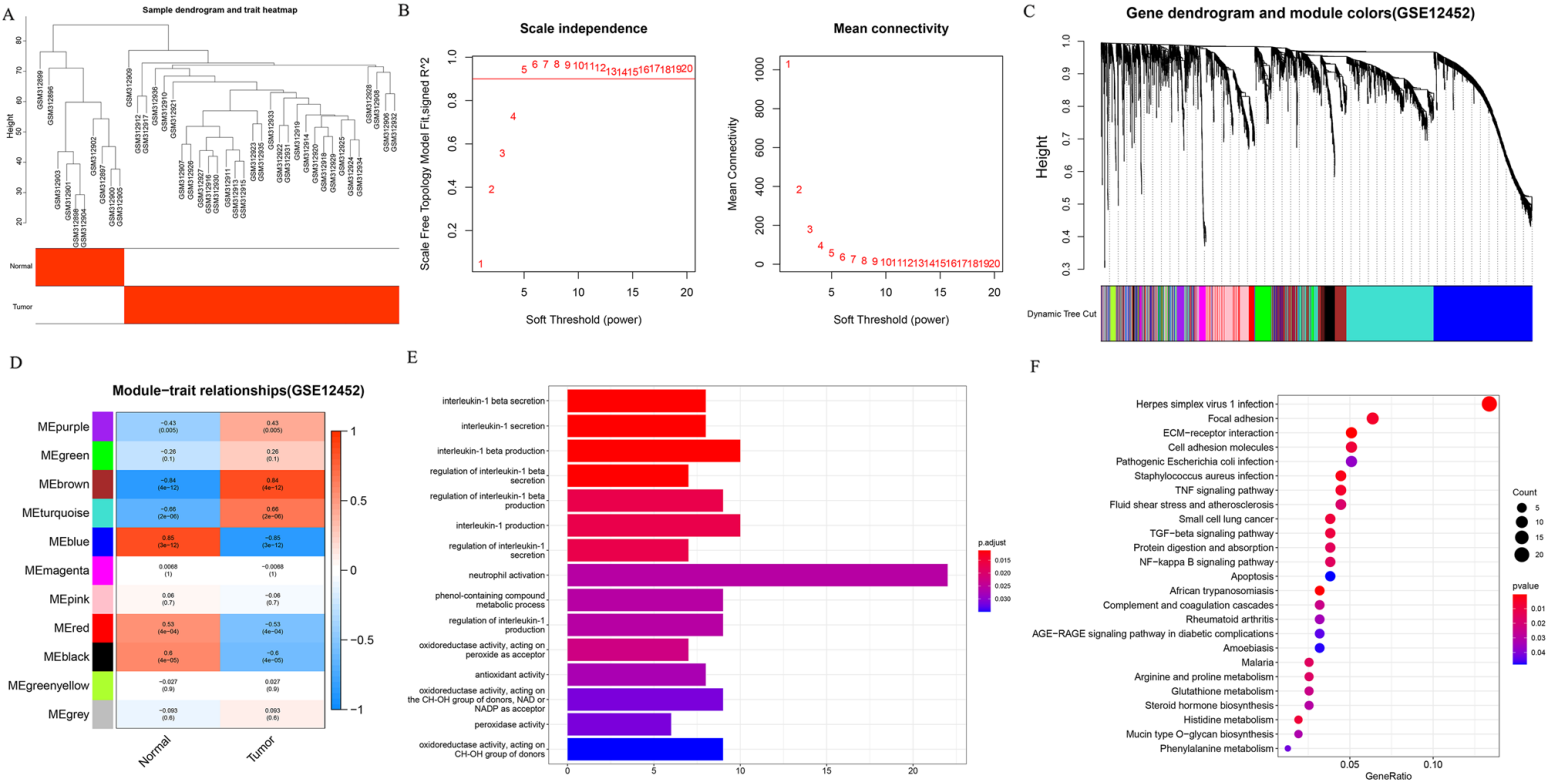
CEP135 is a key miR-26b downstream target gene in NPC. **A** Results of cluster analysis in mRNA dataset GSE12452; **B** A power index of 5 was chosen as a suitable soft threshold; **C** Cluster map of modules obtained after clustering analysis; **D** A module-sample trait correlation heatmap was obtained, with each module containing relevant correlation coefficient and p-value. It was determined that the brown module had the highest correlation coefficient in NPC group and was statistically significant. **E, F** The genes in the brown module were subjected to GO/KEGG pathway analysis

The above study revealed that miR-26b has a crucial function in cell proliferation, migration and apoptosis, but its subsequent pathways and mechanisms need to be further investigated. The original expression matrices downloaded from the GEO database were examined using WGCNA to filter significant genes. The results of the clustering analysis are shown in Fig. 3A. The clustering analysis was performed, and a power index of 5 (Fig. 3B) was chosen as a suitable soft threshold to obtain a modular clustering map (Fig. 3C). A module-sample trait correlation heatmap was obtained, with each module containing the corresponding correlation coefficient and p-value (Fig. 3D). The above results revealed that the brown module had the highest correlation coefficient in NPC group and was statistical significance (*P *< 0.05). The genes of brown module were subjected to GO/KEGG pathway analysis, and the results are depicted in Fig. 3E, F.

### miR-26b Directly Binds to CEP135 and Negatively Regulates the Expression of CEP135

**Fig. 4:**
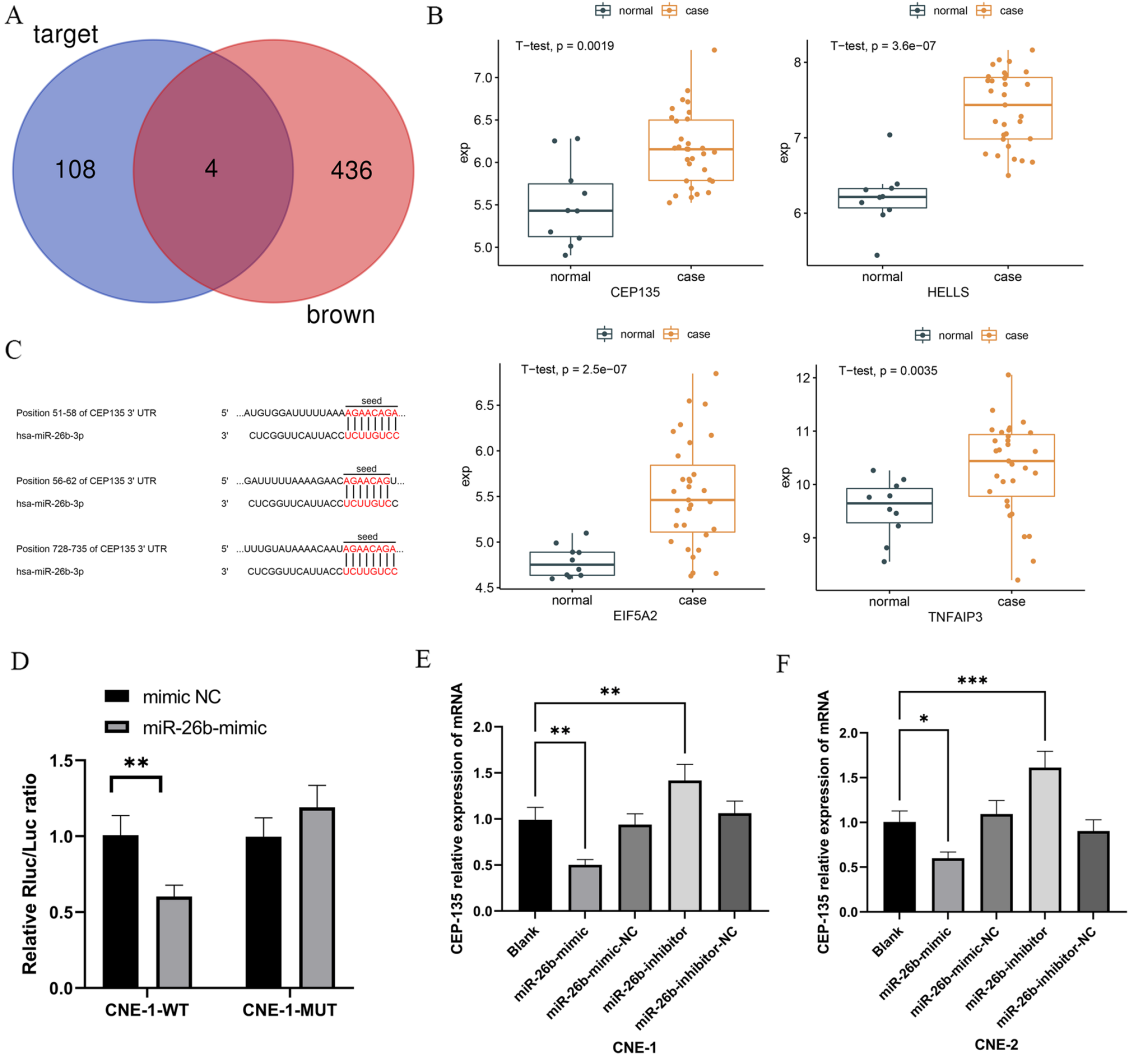
miR-26b directly binds to CEP135 and negatively regulates the expression of CEP135. **A** There are 104 downstream target mRNA of hsa-miR-26b predicted by TargetScan database, Intersection with the brown module fraction obtained from mRNA data analysis was taken to obtain 4 target mRNAs; **B** The differences of 4 target genes in tumor and non-tumor cases; **C** TargetScan database analysis yielded 3 targets of CEP135 and hsa-miR-26b; **D** Results of dual luciferase reporter gene assay; **E, F** The miR-26b-mimic, miR-26b-mimic-NC, miR-26b-inhibitors, and miR-26b-inhibitor-NC were transfected into CNE-1 and CNE-2 cells and RT-qPCR was adopted to detect the CEP135 expression level (*P < 0.05; **P < 0.01; ***P < 0.001)

There are 104 downstream target mRNA of hsa-miR-26b predicted (context +  + score < -0.5) by TargetScan database (http://www.targetscan.org/), and the smaller the context +  + score, the more likely it is the target gene (Schedule 1). Intersection with the brown module fraction obtained from mRNA data analysis was taken to obtain 4 target mRNAs, which were CEP135/TNFAIP3/HELLS/EIF5A2 (Fig. 4A). The R software “ggplot2” package drew box plots of tumor and non-tumor for the four target genes (*P *< 0.05) (Fig. 4B). The hsa-miR-26b target gene was finally identified as CEP135 according to the ranking of context +  + score in Schedule 1. TargetScan database analysis revealed three targets of CEP135 with hsa-miR-26b, including 51–58, 56–62, and 728–735 of CEP135 3′-UTR (Fig. 4C), and CEP135 3′-UTR 728–735 was chosen as the predicted hsa-miR-26b target. The link between CEP135 and hsa-miR-26b was confirmed using dual luciferase reporter gene assays. First, luciferase reporter vectors were constructed, including wild-type and mutant CEP135 3′-UTR. Second, these reporter genes were co-transfected in CNE1 and CNE2 cells with miR-26b-mimic or hsa-miR-NC. The findings demonstrated that, in contrast to the mutant group, overexpression of miR-26b decreased luciferase activity in the wild-type group (Fig. 4D). These findings imply that miR-26b directly binds to the predicted binding location in the CEP135 3′-UTR and inhibits CEP135 production. To further confirm the direct regulation of CEP135 expression by miR-26b, miR-26b-mimic, miR-26b-mimic-NC, miR-26b-inhibitor and miR-26b-inhibitor-NC were transfected into CNE-1 and CNE-2, and RT-qPCR was employed to measure CEP135 expression levels, the results showed that compared to the group transfected with miR-26b-inhibitor-NC and miR-26b-mimic-NC, the expression of CEP135 was significantly decreased in the group transfected with miR-26b-mimic (*P *< 0.05), while in the group transfected with miR-26b-inhibitors, CEP135 expression was considerably elevated (*P *< 0.05) in the group transfected with miR-26b-inhibitors (Fig. 4E, F). All these results indicated that miR-26b directly binds to the predicted binding location in the CEP135 3′-UTR and inhibits CEP135 production.

### CEP135 Affects Cell Biological Behavior Through NF-κB Pathway

**Fig. 5:**
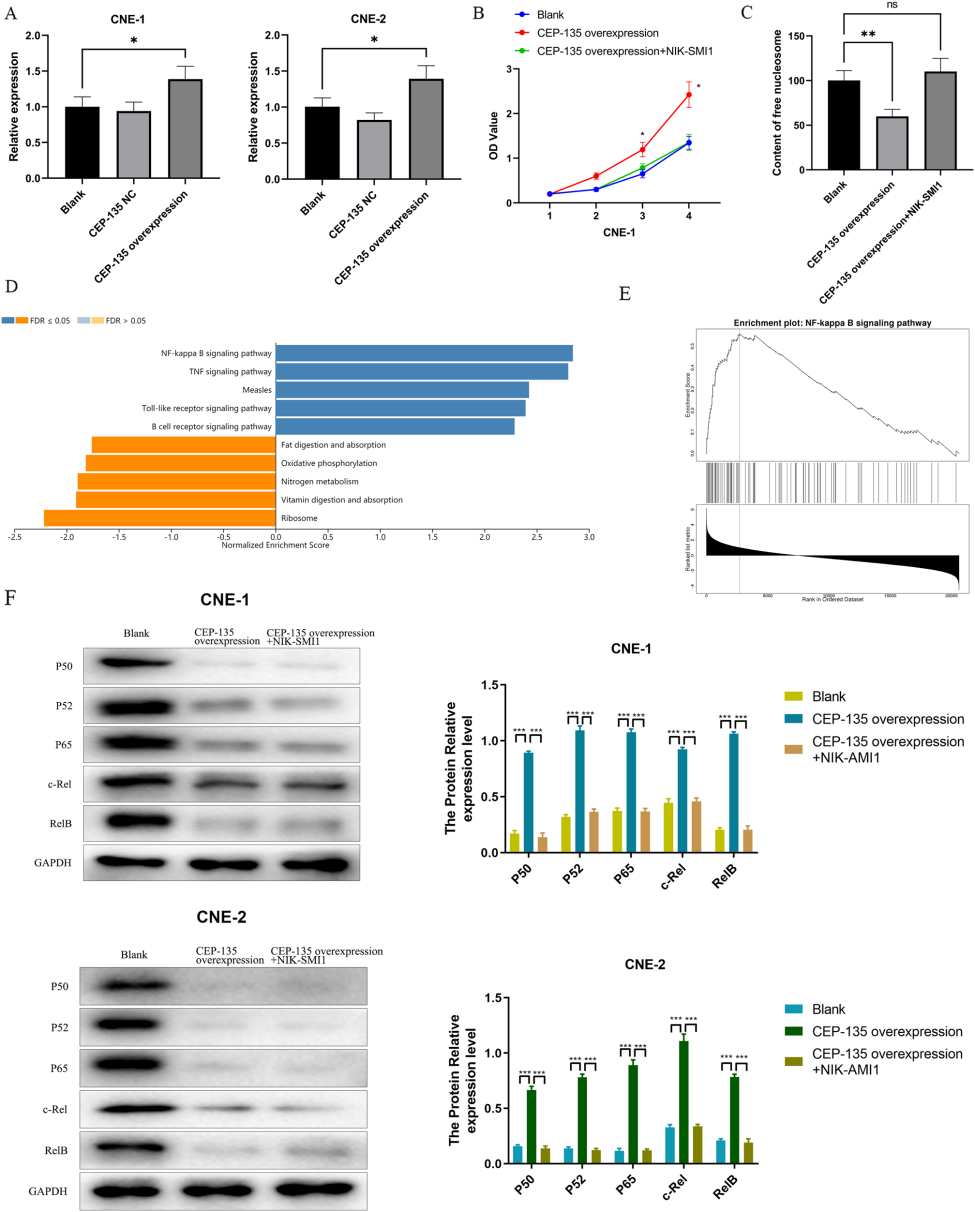
CEP135 gene affects cell biological behavior through NF-κB pathway**. A** Adenovirus strains containing the CEP135 gene and negative control oligomers were transfected into CNE1 and CNE2 cell lines and RT-qPCR was utilized to observe CEP135 expression. **B** The CCK8 test results in three groups; **C** The ELISA test results in three groups; **D** Cancer patients in dataset GSE12452 were separated into two groups, based on the median CEP135 expression: those with high and those with low expression; **E** Bayesian analysis was performed to obtain genome-wide t-values and to plot KEGG pathway enrichment analysis; **F** In both cell lines, NF-κB pathway targets in each group of CEP135 expression and changes in target expression levels after the NF-κB inhibitors is added. Changes in biological behavior of cells in each CEP135 expression group with NF-κB inhibitors in both cell lines (*P < 0.05; **P < 0.01; ***P < 0.001; ns: no significance)

Adenovirus strains containing the CEP135 gene and negative control oligomers were transfected into CNE1 and CNE2 cell lines and RT-qPCR was performed to confirm successful transfection (Fig. 5A). The successfully transfected cells were subjected to cell proliferation and apoptosis analysis.

The CCK8 assay results CEP135 overexpression resulted in significantly faster cell proliferation compared to the blank control, and the addition of NF-κB signaling blocker caused the growth rate of cells to reverse to the original level. (Fig. 5B). The results of ELISA apoptosis kit showed that in contrast to the blank control group, overexpression of CEP135 inhibits the apoptosis, which then reverses to normal levels after the inhibitor is added (Fig. 5C). In conclusion, upregulation of CEP135 expression can further enhance proliferation and prevent apoptosis in tumor cells. On the basis of the median expression of CEP135, secondary analysis was performed on the dataset GSE12452, and cancer patients in the dataset were separated into two groups, high expression and low expression. Bayesian analysis was performed to obtain genome-wide *t*-values, and KEGG pathway enrichment analysis was plotted. The NF-κB pathway was shown to be associated with the gene CEP135 (Fig. 5D, E). The correlation between CEP135 and NF-κB was verified at the cellular level. CNE1 and CNE2 cells transfected with adenovirus containing CEP135 gene were grouped as follows: normal group (blank control), transfected CEP135 overexpression group and CEP135 overexpression group + inhibitor NIK SMI1 group (NIK SMI1: also known as bortezomib, a specific inhibitor of NF-κB). Western blot examined the expression of several important targets on the NF-κB pathway in three groups, including P50, P52, P65, c-Rel and RelB. The findings demonstrated that several targets on the NF-κB pathway were dramatically more expressed in both cell lines in the CEP135 overexpression group than in the blank control group (p-value). In contrast, the multi-target expression on the pathway could be down-regulated to the level of the blank control group after the addition of NF-κB inhibitor. It is suggested that CEP135 regulates the cells through NF-κB pathway (Fig. 5F). The above results showed that CEP135 affected the biological behavior of NPC cells through NF-κB signaling pathway.

## Discussion

In clinical work, distant metastasis of NPC after the first treatment is still a key problem to be solved urgently, and the therapeutic effect of NPC after metastasis is extremely unsatisfactory. For the diagnosis, treatment, and prognostic evaluation of cancers, the study of various biological behaviors such as tumor proliferation, metastasis, and apoptosis is extremely important. Previous studies on NPC have also identified some relevant factors, but no ideal targets have been found yet. With the establishment of tumor database, the collection of big data, and the development of sequencing technology and microarray analysis technology, we will be able to analyze tumor-related information in multiple dimensions, of which miRNA is also an important part. According to recent research, miRNA profiling can classify human tumors more accurately than mRNA profiling, and miRNA-based therapy may be a more promising anti-cancer treatment option. miRNA has now been shown to be useful in the diagnosis, therapy, and prognosis of malignant tumors in both solid and hematologic malignancies [32–35]. An excellent example of miRNA replacement treatment is MRX34, a liposome based on miR-34 mimics, which has become the first miRNA to enter phase I clinical trials (http://clinicaltrials.gov/ct2/show/NCT01829971). As a result, it is particularly critical to identify key miRNAs linked with various biological behaviors of tumors so that relevant targets may be identified for intervention.

In this study, differential analysis was performed on two datasets, GSE32960 and GSE43039, to screen for low expression miRNAs associated with NPC. The intersection of low expression miRNAs from the two datasets was taken and four downregulated miRNA associated with NPC were identified, including hsa-miR-101/hsa-miR-26b/hsa-miR -143/hsa-miR-150, of which the mechanism of action of hsa-miR-101 [36]/hsa-miR-143 [37]/ hsa-miR-150 [38] in NPC has been studied, while the mechanism of hsa-miR-26b in NPC has not been studied. Furthermore, we confirmed that hsa-miR-26b expression was downregulated in NPC cell lines. After downregulating hsa-miR-26b expression in normal epithelial cells, the cells exhibited a propensity to undergo tumor transformation. This evidence suggests that hsa-miR-26b is involved in the various biological behaviors of nasopharyngeal carcinoma. hsa-miR-26b belongs to the hsa-miR-26 family, which includes hsa-miR-26a, hsa-miR-26b, hsa-miR-1297 and hsa-miR-4465, and is a group of extensively conserved small RNAs with identical sequences located in the seed region. Several studies have shown that members of the hsa-miR-26 family have been measured in various malignancies and are lowly expressed in many types of solid tumors. Ji.J’s team examined hsa-miR-26a/b expression in 455 liver cancer tissues and discovered that it was considerably decreased in cancer tissues [39]. It was also reported to be downregulated in esophageal, gastric, and breast cancers [40].

The cellular metabolism, proliferation, differentiation, apoptosis, and metastasis are regulated by the downstream target genes of hsa-miR-26b. The 3′UTR of target genes and miRNA couple up in an imperfectly complementary manner for miRNA to operate. miRNA functions through imperfectly complementary base pairing with the 3′UTR of target genes. hsa-miR-26b has multiple validated oncogenic targets. In bladder cancer, hsa-miR-26b prevents tumor migration and invasion by regulating the PLOD2 gene and it performs a similar function by targeting the SMAD1 gene in liver cancer. In the present study, we also revealed that hsa-miR-26b is down-regulated in tumor cell lines and affects cell expansion, migration and apoptosis. The downstream target gene of hsa-miR-26b was identified as CEP135 by intersecting the predicted target genes from the TargetScan database with the target genes from the database of nasopharyngeal carcinoma mRNA data. The tumor biology was also determined to be affected by hsa-miR-26b through targeting CEP135. This conclusion is based on evidence as follow (1) its overexpression by viral transfection of hsa-miR-26b-mimic significantly reduces the expression level of CEP135 in the corresponding cells; (2) luciferase reporter analysis confirms that hsa-miR-26b can directly bind the 3′-UTL of the CEP135 gene; (3) CEP135 expression was dramatically upregulated in NPC cells and the upregulation was closely correlated with hsa-miR-26b downregulation. However, previous studies on the correlation between hsa-miR-26b and CEP135 were less studied.

CEP135 encodes a centriole assembly protein. Two centrioles make up the centrosome, which replicates once during the S phase when the cell cycle arrives. Once the coupling between centrosome replication and the cell cycle is lost, excess centrosomes are created, a condition known as centrosome amplification. Centrosome amplification promotes cell invasion and chromosomal instability, both of which are characteristics of cancer. chromosomal locus amplification and mutation of CEP135 (4q12) play a significant role in the pathogenesis of aggressive breast cancer [41]. The role of aberrant CEP135 expression in the pathogenesis of NPC, on the other hand, has not been studied. In this study, CEP135 overexpression gene was transfected with virus, which proved that CEP135 could enhance the proliferation and slow down the apoptosis rate of NCP cells.

Finally, using KEGG pathway enrichment analysis, the CEP135 gene was discovered to be linked with the NF-κB pathway, which was verified by cellular studies. In virally transfected CEP135 overexpressed cells, the addition of NF-κB pathway inhibitors significantly downregulated the expression of relevant targets in the pathway, and the cell proliferation rate and apoptosis rate returned to the level of non-overexpressed cells. This suggests that the CEP135 gene is indeed correlated with the NF-κB pathway, but the details remain to be further investigated.

## Conclusions

In conclusion, this study revealed for the first time that hsa-miR-26b was associated with NPC proliferation, migration, and apoptosis. hsa-miR-26b expression was down-regulated in NPC. hsa-miR-26b targets CEP135 gene to regulate proliferation and migration of NPC by NF-κB pathway. These results suggest that hsa-miR-26b may provide a new idea as anti-tumor target for the treatment of NPC.

### Supplementary Information

Below is the link to the electronic supplementary material.

Supplementary file1 (xlsx 14 KB)
